# Developmental characteristics of pearl oyster *Pinctada fucata martensii*: insight into key molecular events related to shell formation, settlement and metamorphosis

**DOI:** 10.1186/s12864-019-5505-8

**Published:** 2019-02-08

**Authors:** Zhe Zheng, Ruijuan Hao, Xinwei Xiong, Yu Jiao, Yuewen Deng, Xiaodong Du

**Affiliations:** 1 Fishery College, Guangdong Ocean University, Zhanjiang, 524088 China; 2 Pearl Breeding and Processing Engineering Technology Research Center of Guangdong Province, Zhanjiang, 524088 China

**Keywords:** Shell formation, Settlement, Metamorphosis, Molecular events

## Abstract

**Background:**

Marine bivalves undergo complex development processes, such as shell morphology conversion and changes of anatomy and life habits. In this study, the transcriptomes of pearl oyster *Pinctada fucata martensii* and Pacific oyster *Crassostrea gigas* at different development stages were analyzed to determine the key molecular events related to shell formation, settlement and metamorphosis.

**Result:**

According to the shell matrix proteome, biomineralization-related genes exhibited a consensus expression model with the critical stages of shell formation. Differential expression analysis of *P. f. martensii*, revealed the negative regulation and feedback of extracellular matrixs as well as growth factor pathways involved in shell formation of larvae, similar to that in *C. gigas*. Furthermore, neuroendocrine pathways in hormone receptors, neurotransmitters and neuropeptide receptors were involved in shell formation, settlement and metamorphosis.

**Conclusion:**

Our research demonstrated the main clusters of regulation elements related to shell formation, settlement and metamorphosis. The regulation of shell formation and metamorphosis could be coupled forming the neuroendocrine-biomineralization crosstalk in metamorphosis. These findings could provide new insights into the regulation in bivalve development.

**Electronic supplementary material:**

The online version of this article (10.1186/s12864-019-5505-8) contains supplementary material, which is available to authorized users.

## Backgrounds

Bivalves comprise millions of species that are widely distributed in the world as common benthic community in coastal areas. Marine bivalves undergo complex life development processes, such as shell morphology conversion as well as changes of anatomy and life habits [1]. At these stages, larvae are sensitive and perishable. Many endogenous physiological processes and exogenous physical and chemical elements could affect their survival.

Bivalve shells are the typical biomineralization products, with multiple morphological structure and special stress. The symbolic bivalves’ shells covering the body surface in both larvae (the protoconch) and adults stages (the dissoconch), even with different calcium carbonate crystal products, could function on mechanical stability, physical protection and even chemical defence [2, 3]. As the shells of bivalves undergo the big change in the development stage, such as the crystal polymorph transition that happens need special physical conditions in nature, the formation and regulation mechanisms of bivalve shells are important to the development of biomaterials. And the mechanisms by which larvae shells are fabricated and conversed in the development processes along with the morphological metamorphosis are important to understand the evolution and regulation of mineralization in biological organisms [4]. Complex organic macromolecules compounded by mantle tissue cells harnessing the processes of nucleation along with haemocyte have been developed for mediating the process of shell formation [5, 6]. Recently, the main biomolecular components in several shellfish, such as bivalves and gastropods species, have been identified [7–11]. Some regulation pathways and transcriptional factors involved in shell formation have been elucidated [12–14]. Most of them are homologues of regulators in the operation of endoskeleton system construction. However, the vital regulation factors in shell morphology construction remain unclear.

Metamorphosis of bivalve species is a significant physiological process from free-living larva to sessile juvenile that includes loss of the velum, development of tissues, such as gills and foot, and production of adult shell [15]. The gradual covering of protoconch accompanied with the veliger stage, which represents the changes of digestive and swimming behaviors. Adult dissoconch is one of the indicators of the transition of free-swimming larvae to benthic and often sessile and attached juveniles. And larvae at this stage are sensitive to the environment and easy to die. In bivalves, larval responses to environmental cues, including salinity, depth, temperature and light, and the main biophysical cues, such as neuroendocrine compounds and appropriate ion concentrations, influence their subsequent settlement and metamorphosis. Recently, specific neurotransmitters and chemicals have been found to induce specific responses in initiating bivalve metamorphosis [16–18]. As many of bivalve species are the important economic species such as oysters, finding the regulation mechanisms of metamorphosis in bivalves could contribute to improve the rate of survival and promote relative industries. In addition, several neuroendocrine factors and pathways across invertebrate and vertebrate species are evolutionarily conserved and ancient origins [19]. While, recently, a series of artificial inducers, such as neurotransmitters and analogues used as pesticide or anticorrosive paint, act as marine pollutants and influence larval settlement and metamorphosis of marine invertebrates [20]. Therefore, exploring the intrinsic neuroendocrine pathways in bivalves could contribute to marine environmental protection and species conservation.

In this study, we selected pearl oyster *Pinctada fucata martensii* as well as Pacific oyster *Crassostrea gigas* with high precision genomic data, intact developmental transcriptome data and shell matrix proteome [21, 22]. These data were reanalyzed to explore the key molecular events related to shell formation, settlement and metamorphosis*.* We also determined the important molecular events involved in shell formation and proposed the neuroendocrine-biomineralization crosstalk in metamorphosis.

## Methods

### Collection of supported data of the pearl oyster *P. f. martensii* and the Pacific oyster *C. gigas*

The genome data and transcriptome of *P. f. martensii* at different development stages and in different tissues, as well as the shell matrix proteome data were downloaded from GigaDB (http://gigadb.org/search/new?keyword=Pinctada+fucata+martensii). The transcriptome and genome data of *C. gigas* at different development stages and its shell matrix proteome data were downloaded from https://www.nature.com/articles/nature11413, supplementary tables [21].

### Different expression analysis and function enrichment of *P. f. martensii* at different development stages

Target genes (TGs) that differently expressed with the condition (|log2 Ratio| > 1, FDR < 0.001) were selected for the function enrichment at different development stages. We performed functional enrichment analysis of our TGs with the commonly used Gene Ontology (GO) and KEGG databases. GO provides three ontologies: molecular function, cellular component and biological process. By comparing with the background of all genes, enrichment analysis provides all terms (GO term, pathway ID) that are significantly enriched in the TGs. We developed a strict algorithm for the analysis, with *p*-value defined as:

$$ \mathrm{P}=1-\sum \limits_{i=0}^{m-1}\frac{\left({}_i^M\right)\left({}_{n-i}^{N-M}\right)}{\left({}_n^N\right)} $$

Where N is the count of all genes with functional annotation; n is the count of TGs in N; M is the count of all genes that are annotated to certain functional terms; and m represents the count of TGs in M. The calculated p-value was subjected to Bonferroni Correction, taking corrected p-value ≤0.05 as the significantly enriched. Functional terms fulfilling this condition were defined as significantly enriched functional terms in TGs.

### Identification of specifically expressed genes in mantle edge (ME) and mantle pallial (MP)

According to the published transcriptome data [22], we selected the specifically expressed genes that were highly expressed in one tissue (ME or MP), compared to all other tissues including in the adductor muscle, hemocyte, foot, gill, gonad, hepatopancreas, with criteria that reads per kilobase of exon per million fragments mapped (RPKM) ratio of ≥4 fold (upregulated) and false discovery rate (FDR) of ≤0.05.

### Identification of gene expression patterns

Given that the expression of different genes in the different samples showed considerable difference, we normalized each gene in all samples. Based on normalized expressed values of genes, we used hierarchical clustering of R package with default parameters to identify the gene families with the same expression patterns, and then divided the gene tree into a series of sub-trees according to the expressed similarity in expression patterns between genes.

## Results

### KEGG and GO enrichment analyses of differentially expressed genes among critical development stages

**Fig. 1:**
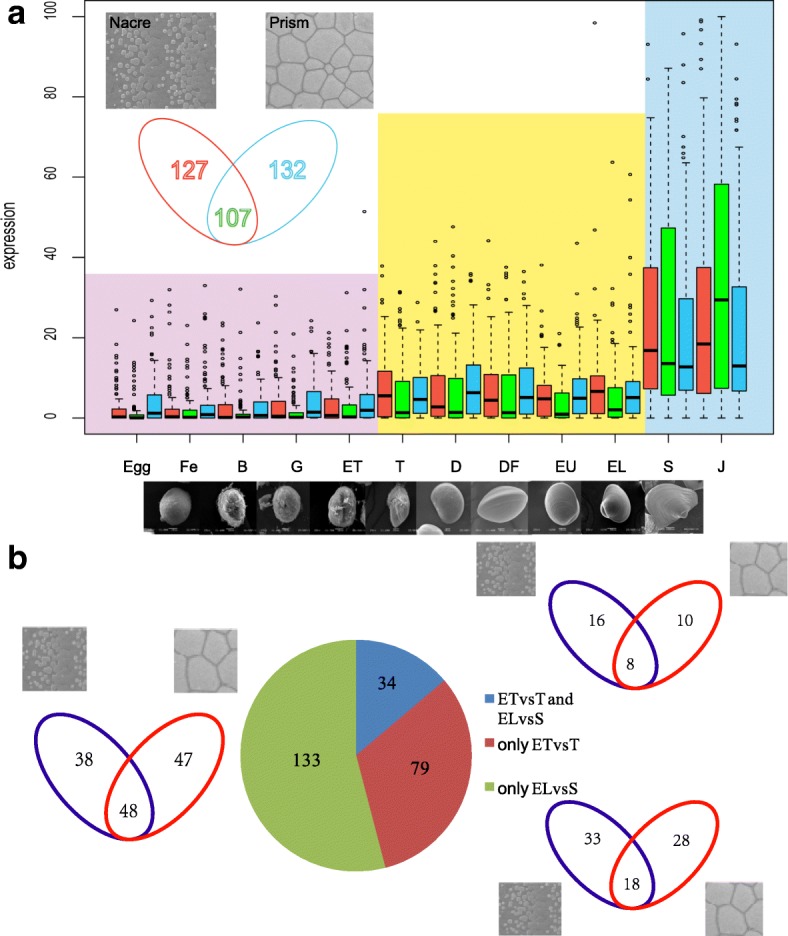
Genes coding for shell proteins expression model at different development stage in *P. f. martensii.* **a**. the expression pattern of shell protein coding genes at different development stage in *P. f. martensii*. The y-axis is the normalized RPKM value. Egg, egg; Fe, fertilized egg; B, blastula; G, gastrula; ET, early trochophore; T, trochophore; D, D-stage larvae; DF, D-stage larvae before feeding; EU, early umbo larvae; EL, eyed larvae; S, spat; J, juveniles. **b**. the number of differentially expressed genes at the critical stages of shell formation

Basing on the morphological characteristics of larva of *P. f. martensii* at different development stages, trochophore stage (secreted protoconch) and spat stage (secreted dissoconch) were considered as the turning points for shell formation. Moreover, the considerable change of life habits happens from eyed larvae to spat for the transmission from plankton to demersal. To investigate the development pattern, differentially expressed genes were filtered for the contiguous comparison among twelve stages (Fig. 1). To disclose the related metabolic and signaling pathways and functional proteins involved in prodissoconch/dissoconch deposition, we comparative analyses of four groups: early trochophore (ET) vs trochophore (T), trochophore (T) vs D-stage larvae (D), early umbo larvae (EU) vs eyed larvae (EL), eyed larvae (EL) vs spat (S). The results of enrichment of differentially expressed genes based on Cellular Component Gen Ontology (GO) and Kyoto Encyclopedia of Genes and Genomes (KEGG) analyses in each group are shown in Additional file 1 and Additional file 2.

### Consensus analysis of biomineralization-related gene expression and shell formation

In a previous study, we identified 366 proteins from decalcified shell matrix [22]. Those proteins were classified into three groups in accordance with positions: 127 nacre-specific proteins, 132 prism-specific proteins, and 107 proteins in both nacre and prism [22]. To determine the vital stage of shell formation, the expression profiles of those target genes were detected at different development stages. Three groups of shell matrix protein (SMP) genes showed two upregulation points, trochophore stage (T) and spat stage (S) (Fig. 1a). The formation of protoconch appears at the trochophore stage. At this stage, a total of 113 SMP genes expression increased significantly. The laminated nacre and prismatic-calcite layer began to deposit at the spat stage, and also performed the transition from swimming to sessile life. Therefore, it is the crucial period for biomorphological and physiological transformation, especially for disconch formation [1, 2]. We found that the remarkable upregulation of shell proteins happened at the spat stage, which suggested that the SMPs involved in dissoconch formation were obviously different from those for protoconch in *P. f. martensii*. We noticed that 49 nacre-specific genes were significantly up-regulated at trochophore stage, which was similar to 54 at spat stage. By contrast, prism-specific genes (57 genes) and common genes (56 genes) that mostly increased in post-veliger stage were obviously expressed more than those (38 prism-specific genes and 26 common genes) at trochophore stage (Fig. 1b).

We also analyzed the SMPs in oyster *C. gigas* whose shell consisted of foliated calcite due to the presence of “calcite tools” [21]. The expression model of SMPs genes showed three main upregulation points (from T1 and from S) that were matched to the trochophore stage (T) and spat stage (S) in *P. f. martensii*, along with a transitional period appeared at P1 and P2 stages (Additional file 3). The expression models of these genes in *C. gigas* were similar to that in *P. f. martensii*.

**Fig. 2:**
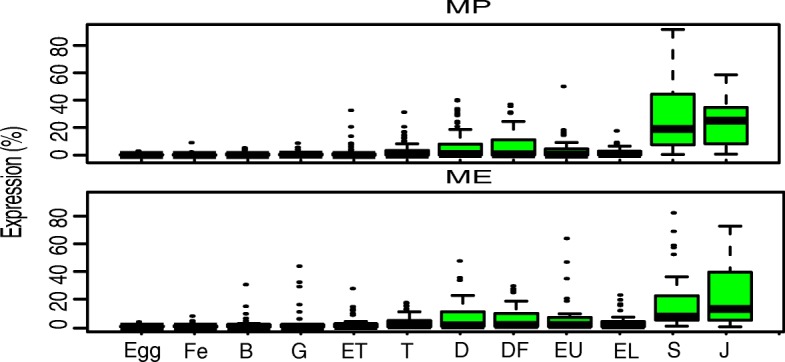
Mantle tissue specific genes of *P. f. martensii*. ME, mantle edge; MP, mantle pallial

Mantle tissue in bivalves is involved in layered and subtle shell formation in adults [23]. The morphogenesis of mantle tissue is accompanied by expression of mantle-specific genes that begin to expression, represents the “turn on” of biomineralization. In this study, we identified a total of 113 mantle-specific genes including 45 mantle-edge-specific (ME) genes and 68 mantle-pallial-specific (MP) genes. Known biocalcification related genes such as tyrosinase and VWA domain containing proteins were included (Additional file 4). The significant upregulation of some ME- and MP- specific genes at D-shape stage also suggested the early morphogenesis of larva bio-calcifying tissue (Fig. 2). Similar to that in *C. gigas* [21], most of the ME- and MP- specific genes were significantly enhanced at the post-veliger stage when dissoconch was formed, corresponding to the histological observations, thus indicating a functional difference of mantle tissue between D-shape larva and the post-metamorphic larva.

### Downregulation and feedback of extracellular matrixes (ECMs)

The hypothesis that organisms sculpt mineralized skeletons via “anti-calcifying” macromolecules because of spontaneous calcification of cell and tissue surface in highly oversaturated Proterozoic oceans, implies that the regulation of dissolution of calcification for livings may be ancient and conserved [24]. In this research, we disclosed the elements belonging to osteoclast differentiation pathway that participate in the downregulation in bone which were enriched at trochophore stage (Additional file 1). As a result, the osteoclast-specific genes involving TRACP (tartrate-resistant acid phosphatase) and CTR (calcitonin receptor) were highly upregulated at the trochophore stage (Additional file 5). The existence of osteoclast differentiated elements in pearl oyster mentioned above implied that the appearance of decalcifying functions accompanied with fabrication of prodissoconch may be the shadow of ancient biomineralization in bivalves, which support the “anti-calcifying” origin of biomineralization. However, the absence of homologues of the vital signal RUNKL and its receptor RUNK as well as the presence of OPG in vertebrate also imply the different regulation system.

**Fig. 3:**
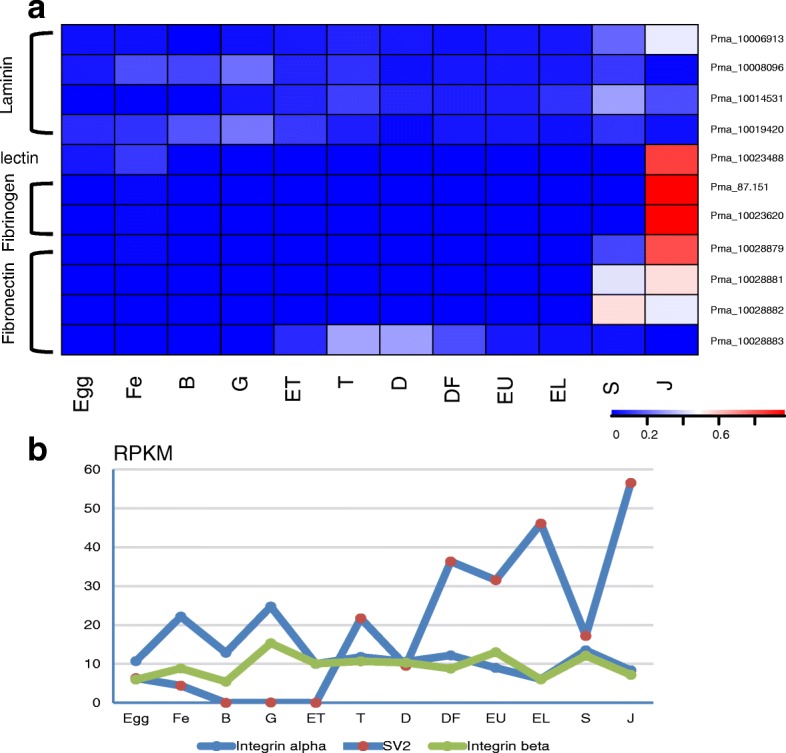
Expression pattern of ECMs in shell matrix (**a**) and the receptors (**b**) at different development stages in *P. f. martensii.* Egg, egg; Fe, fertilized egg; B, blastula; G, gastrula; ET, early trochophore; T, trochophore; D, D-stage larvae; DF, D-stage larvae before feeding; EU, early umbo larvae; EL, eyed larvae; S, spat; J, juveniles

The apparatus of cell junctions and the ECM are critical for every aspect of the organization, function, and dynamics of multicellular structures. A series of SMPs are homologues of ECMs or contain the typical domains [25]. As the components of shell matrix, these proteins could directly participate in shell fabrication. In close association with the surfaces of cells that produce them, ECMs have an active and complex role in regulating the behavior of cells. In this study, the ECM-ligands interaction pathway was significantly enriched from eyed larvae to spat (Additional file 1). The shell matrix proteins such as laminin, fibronectin-like protein, fibrinogen-like, lectin and the reported collagen-like VWA containing proteins [22] showed obviously different expression patterns at trochophore/spat stages in *P. f. martensii* and *C. gigas*, as well as the receptors such as integrin and SV2 (Fig. 3 and Additional file 6). Moreover, the molecular structures of several ECMs such as fibronectin/collagen-likes were different and divergent to the vertebrate homologues.

### Growth factor pathways involved in shell formation of larvae

**Fig. 4:**
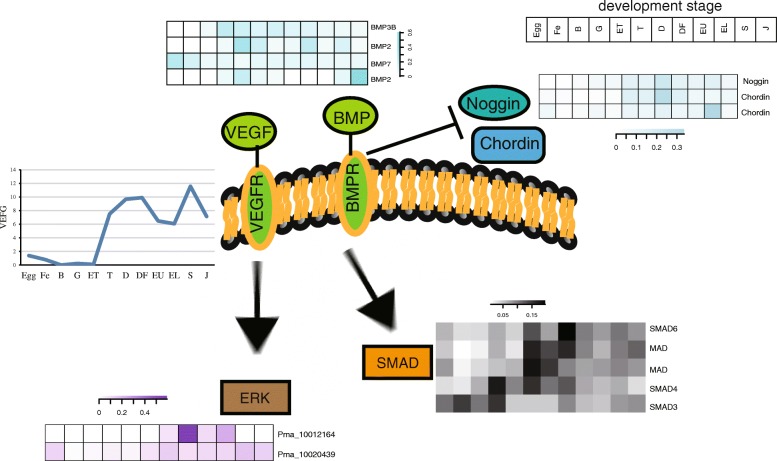
Expression pattern of key elements in VEGF\TGF\ ERK pathways at different development stages in *P. f. martensii.* The y-axis is the normalized RPKM value. Egg, egg; Fe, fertilized egg; B, blastula; G, gastrula; ET, early trochophore; T, trochophore; D, D-stage larvae; DF, D-stage larvae before feeding; EU, early umbo larvae; EL, eyed larvae; S, spat; J, juveniles

We screened the main elements of reported vital growth factor pathways in vertebrates and invertebrates. In pearl oyster and other bivalves, the typical TGF-beta was absent but the main growth factor bone morphogenetic protein (BMPs) and other elements in this pathway were found. We analyzed the expression profiles and found that mainly co-smad 1/5/8 significantly increased as well as the inhibited smad6/7 were upregulated in the trochophore stage. Meanwhile, the inhibitor of TGF-beta/BMP signal such as Noggin and Chordin were also enhanced (Fig. 4). Thus, the simplified and centralized approach to biomineralization regulation was chosen in bivalves. In the development of *P. f. martensii*, VEGF pathway was significantly enriched as the elements obviously increased in trochophore stage (Additional file 1), in especial VEGF, which was consistent with the extensive upregulation of shell protein encoding genes in these two species (Fig. 4). In addition, mantle-specific genes showed apparent expression in subsequent D-shape stage, denoting the initial morphogenesis. Moreover, one ERK gene (Pma_10020439), the downstream elements of VEGF signal pathway, was highly expressed in the trochophore stage, which was characterized as maternal ERK exhibiting high expression level in larval and adult (Fig. 4). Another ERK (Pma_10012164) was mostly expressed in whole veliger larva stages, along with protoconch formation (Fig. 4). MAPK signal pathway was significantly enriched in the different expressed genes between early trochophore stage and trochophore stage. These results indicated that MAPK signal pathway is probably involved in larvae shell formation.

### Neuroendocrine pathways involved in shell formation, settlement and metamorphosis

**Fig. 5:**
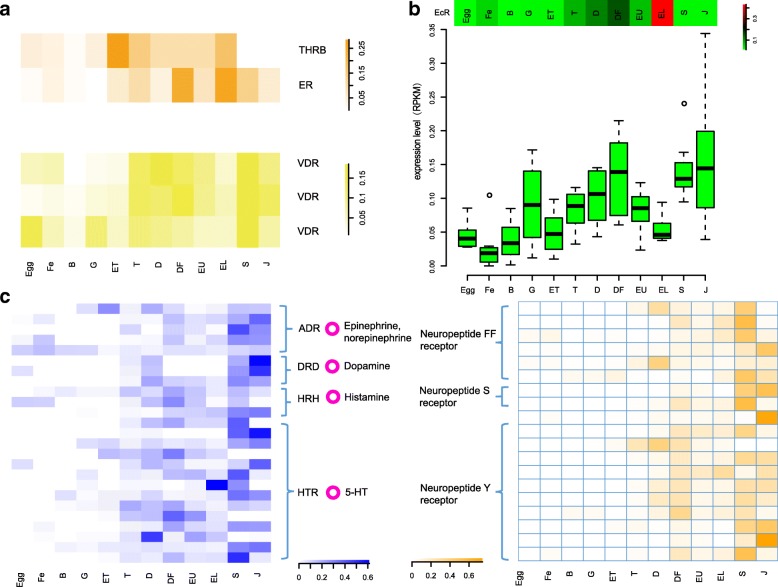
Differentially expressed elements of neuroendocrine pathways involved in shell formation, settlement and metamorphosis. The expression pattern of thyroid hormone receptor (ThR), estrogen receptor (ER) and vitamin D3 receptor (VDR) at different development stages were shown in **a**. The expression pattern of ecdysone receptor (EcR) and other elements in ecdysone signal pathway at different development stages were shown in **b**. Neurotransmitter receptors including adrenergic receptor (ADR), dopamine receptor (DRD), histamine receptor (HRH) and 5-HT receptor (HTR), and neuropeptide Y/S/FF receptors were shown in **c**. The y-axis is the normalized RPKM value

Hormones are involved in embryonic development, homeostasis and physiologically regulated processes including settlement and metamorphosis by regulating the expression of specific genes, via the ligands-receptors recognition and related signal transduction, such as lipid hormone interaction with nuclear receptors (NRs). In this study, 59 differently expressed genes between eyed larvae and spat were included in 12 process categories. The process categories of GO enrichment include “response to chemical stimulus”, and “response to endogenous stimulus” (Additional file 2). We disclosed the included genes and their expression profiles at different development stages. Several hormone receptors closely related to bone formation in vertebrate were found in the enrichment group containing thyroid hormone receptor (ThR), estrogen receptor (ER) and vitamin D3 receptor (VDR) (Fig. 5a).

Notably, we found that several elements involved in ecdysone signal pathway represented significantly different expression before metamorphosis (Fig. 5b). We screened the genomic data and successfully identified the ecdysone receptors (Ecrs) in *P. f. martensii* and *C. gigas* and other bivalve species (Additional file 7a). The expression pattern of Ecr in *C. gigas* was consistent with that in *P. f. martensii* (Additional file 7b). The biosynthesis of ecdysone from cholesterol in insects needs several synthases, including cholesterol 7-dehydrogenase (Nvd), and several cytochrome P450s. We firstly identified the Nvd homologue and found one (Pma_10025862) in the genome of *P. f. martensii* as well as one homologue genes in *C. gigas* (CGI_10020287) showed the similar expression trend with an upregulation before metamorphosis (Additional file 7c). The expression levels showed similar. We also found that *P. f. martensii* possessed some of the elements that are necessary for signal transduction of ecdysone and showed significantly different expression patterns from early umbo larvae to eyed larvae and to spat stages (Fig. 5b). Thus, we proposed that bivalve species possessed the relative elements in hormone signal pathway and could play vital roles in shell formation.

In addition, we found that neuroactive ligand-receptor interaction was significantly enriched in the differently expressed genes between eyed larvae and spat (Additional file 1). Tyrosinase metabolism could trigger L-DOPA, which is the precursor to the neurotransmitters DA, NE and EPI that were significantly enrichment between EL and S. The receptors of acetylcholine, epinephrine, dopamine, histamine, 5-HT and neuropeptide Y/S/FF receptors that belonged to GPCRs were obviously upregulated at the trochophore stage and/or spat stage in pearl oyster (Fig. 5c, Additional file 8). These results indicated that neuroendocrine system play critical roles in settlement and metamorphosis including shell formation.

## Discussion

In the development processes of pearl oyster and other benthic marine bivalves, the change in life habit from plankton to the bottom settlement is their remarkable character. This difference could be related to the adaptation to their habitat. The loss of the freely swimming ability indicated the weakness of initiative eluding defence. The primary shells protoconch that appear from trochophore larva could protect larvae’s body to some extent. However, adult shells with anti-stress microstructure are formed instead of protoconch in veliger larva; the laminated nacre and prismatic-calcite layer deposited at spat stage, are for physical and chemical protection [26]. Therefore, the spat stage is the crucial period for biomorphological and physiological transformation, especially for the dissoconch formation [1, 2]. Liu et al. reported the expression profiling during larval development for pearl oyster *P. f. martensii* by microarray, in which the development stages corresponded to the turning points [27]. In this study, we used the SMP genes that directly participated in shell formation for the consensus analysis in *P. f. martensii* and *C. gigas*, because these two species had multiple development transcriptome data among bivalves. The massive upregulations of shell proteins happened at spat stages which suggested that the SMPs included in dissoconch formation were obviously different from those for protoconch biomineralization in *P. f. martensii*. Nacre-specific genes were significantly upregulated at trochophore stage, but the number of prism-specific and common genes that mostly increased at post-veliger stage were obviously more than those in trochophore stages. Thus, the proteins closely related to aragonite calcification were expressed in the early protoconch formation, whereas the proteins responsible for calcite prismatic layer formation and those for both types of calcifications were later expressed at the spat stage. This finding was consistent with the study of Naoki Yokoo et al., in which the embryo was covered with the first shell made of aragonite with the c-axis normal to the shell [2]. We also analyzed the SMPs in oyster *C. gigas* whose shell consisted of foliated calcite, which was proposed to be due to the presence of “calcite tools” [21]. The expression model of SMPs genes showed two main upregulation points (trochophore and spat), which were matched with trochophore stage and post-veliger stage in *P. f. martensii* and divided the development processes into three stages, along with a transitional period at the P1 and P2 stages. In addition, the crystalline form of protoconch of *C. gigas* is aragonite [28]. That is to say, part of the “calcite tools” in *C. gigas* were executor in the early aragonite protoconch formation. The findings suggested that, to some extent, the “calcite tools” and “aragonite tools” were not strictly distinct, which explained the existence of abundant common proteins in both prismatic layer and nacreous layer in the shell of pearl oyster. The transformation of crystalline form of calcium carbonate may not only depend on decisive function proteins, but also to the proportions of functional proteins or other conditions. In addition, the appearance of a fragile, prismatic layer accompanied with the lamellar layer in Pteria, also indicated that the appearance of lamellar morphological structure may be actuated for remedying the vulnerability of the formed prismatic morphological structure.

The hypothesis that organisms sculpt mineralized skeletons via “anti-calcifying” macromolecules for spontaneous calcification of cell and tissue surface in highly oversaturated Proterozoic oceans, implied that the regulation of dissolution of calcification for livings may be ancient and conserved [24]. Several matrix protein families involved in osteogenesis of calcium phosphate were gradually incorporated in the exoskeleton of calcium carbonate, which suggested the common ancestor of biomineralization [29]. Nonetheless, there is a little evidence to illustrate the negative regulation mechanism of biomineralization. In this study, the existence of homologues of osteoclast differentiated elements such as TRACP and CTR [30, 31] in pearl oyster, and the apparent upregulation at trochophore implied that the appearance of decalcifying functions accompanied with generation of prodissoconch may be the shadow of ancient biomineralization in bivalves, thereby supporting the “anti-calcifying” origin of biomineralization. However, the absence of homologues of the vital signal RUNXL and its receptor RUNX as well as the presence of OPG in vertebrate also indicated the different regulation systems [32].

Another feedback control on the regulation of shell formation may reflect on the ECM-ligand interaction. The ECM consists of a complex mixture of structural and functional macromolecules and serves an important role in tissue and organ morphogenesis and in maintenance of cell and tissue structure and function. ECMs could control cell differentiation and gene expression through adhesive interactions [33]. This research showed that the dramatic change of bivalves in metamorphosis exhibited the different expressions of ECMs and their receptors. The interactions of ECM and their receptors lead to a direct or indirect control of cellular activities [34]. In shell matrix, some components were homologues of ECMs in mammals such as collagen-like VWA domain containing proteins, fibronectin-like proteins and laminins [10, 22]. These findings suggested that ECM in shells may have independently evolved and exhibited novel functions that needs to be further illustrated. In addition, other components of shell matrix proteins also have potentially feedback to calcification-related tissues. The cellular model shows that shell formation is orchestrated by cells, and the ECM and crystals are formed in haemocytes in eastern oyster, *Crassostrea virginica* [35]*.* Fabien Badariotti et al. found that the chitinase-like protein from the oyster *C. gigas* can promote cell proliferation and regulate ECM component synthesis [36]. Therefore, we proposed that the ECM components had the capacity to feedback regulate calcification-related tissues and affect the processes of biomineralization.

In addition, a series of conserved regulation mechanisms related to biomineralization and skeleton/exoskeleton formation were found in this study. The VEGF\TGF\ ERK/MAP kinase pathways are important mediators of mechanical and hormonal signals in bones [37]. In a previous study, pearl oyster and other bivalves, the typical TGF-betas were absent but the main growth factors, bone morphogenetic protein (BMPs), and other elements in this pathway were found [22]. We found that mainly co-smad 1/5/8 were significantly increased and the inhibited smad6/7 as well as the inhibitor of TGF-beta/BMP signal were upregulated at trochophore, which indicated that the simplified and centralized approach to biomineralization regulation was chosen in bivalve. Moreover, the vascular endothelial growth factor (VEGF) functions in maintaining the homeostasis of the skeletal system, dental healing and kidney disease [38]. In sea urchins, certain ectoderms release signaling cues, such as VEGF, to direct the skeletogenic cells to position and secret the skeleton consisting of calcite rods [39, 40]. During the development of *P. f. martensii*, VEGF pathway was significantly enriched at the trochophore, especially VEGF, which was consistent with the extensive upregulation of shell protein encoding genes. Moreover, ERK, as the downstream elements of VEGF signal pathway was also highly expressed, which was characterized as maternal ERK exhibiting high expression level in larvae and adults. ERK was also highly expressed at the veliger larva stage, during protoconch formation. Therefore, we proposed that ERK maybe the core effector of the signal pathways involved in the regulation of shell formation. ERK/MAP kinase pathway is an important mediator of mechanical and hormonal signals in bones [37]. The downstream transcriptional factors of MAPK signal pathway, such as c-fos/c-jun, not only function in vertebrate but in bivalve shell formation through upregulating the promoter activation of several shell matrix proteins, such as N19, Prisilkin39, Pearlin, and KRMP [12]. In the present study, the enrichment of MAPK signal pathway suggested that MAPK may be a core pathway for signal transduction, and the correlated signals to the MAPK signaling pathway could directly or indirectly participate in the regulation of shell formation in bivalves. These findings supported the conserved regulation mechanisms among bivalves and vertebrates, which contribute the understanding of the mechanism of bionic materials of shells in bone repair.

Shell fabrication in bivalves are important part in during settlement and metamorphosis. Moreover, the neuroendocrine system plays critical roles in settlement and metamorphosis, and neurotransmitters and neuropeptides have an early evolutionary origin and are involved in cell differentiation, development and morphogenesis in marine invertebrate [20, 41, 42]. In this study, we found that several neuropeptide receptors such as neuropeptide Y/FF/S, were upregulated at the trochophore or spat stages in *P. f. martensii* and *C. gigas.* The biosynthesis of some neuroactive compounds, such as the tyrosinase metabolism, could trigger L-DOPA the precursor to the neurotransmitters DA, NE and EPI, which were significantly enriched [20]. Tyrosine is also involved in the biosynthesis of other neurotransmitters as a precursor for tyramine and octopamine (OA), which are known primary neurotransmitters in other invertebrates [43]. Several receptors of neuroactive compounds also exhibited the upregulated expression when metamorphosed in this study. These results supported the critical roles of neuropeptides in the settlement and metamorphosis of bivalves, but further study involving direct detection of neuroactive compounds is needed.

Furthermore, the differentially expressed genes between eyed larvae and spat suggested an apparent functional difference in hormone regulation. The enrichment of endogenous stimulus categories, including several hormone receptors closely related to bone formation in vertebrate such as ThR, ER and VDR [44] showed significantly different expression from veliger to spat in *P. f. martensii* and *C. gigas*. These findings indicated the connections between bivalves and vertebrates in terms of biomineralization regulation controlled by endocrine and the crosstalk regulation in metamorphosis and biomineralization.

In addition to the conservation to vertebrates, we also found the important hormone Ec controlling the metamorphosis in arthropod and its receptor Ecr, along with other elements in its pathway, which exhibited an obviously change before and after settlement and metamorphosis. In arthropods, the ecdysone pathway controls a series of complex biogenic activities in developmental processes, such as moulting exoskeleton, through regulating the synthesis and degradation of the main component chitin, as well as activating a series of changes for metamorphosis [45, 46]. Chitin is also considered the base membrane in shell organic matrix [47]. The biosynthesis of ecdysone from cholesterol in insects needs several synthase, including Nvd and several CYP450 that participates in the transformation of cholesterol to ecdysone and 20-hydroxyecdysone [48, 49]. In the study, we found the critical Nvd homologue in *P. f. martensii.* The expansion of Cytochrome P450 family was observed, with a total of 491 genes belonging to P450 family members in pearl oyster, but the high conservation to the vital CYPs in molting hormone biosynthesis in insects is lacking [50]. Thus, bivalves could be very likely self-biosynthesize of ecdysone derived from cholesterol using CYPs, but the effect of CYP should be carefully considered. In human, ecdysone stimulates the expression of BMP2 mediated by ERK in human periodontal ligament stem cells [51]. Thus, we proposed that bivalve species possessed the relative elements in hormone signal pathway that could play vital roles in the neuroendocrine-biomineralization crosstalk during metamorphosis. The regulation of biomineralization and settlement and morphology could be coupled with the same neuroendocrine signals.

## Conclusion

Transcriptome analysis of *P. f. martensii* and *C. gigas* showed that multiple molecular events participated in shell formation, settlement and metamorphosis in bivalves. Conserved regulation elements, such as growth factor pathways, negative regulation and feedback of ECMs, were found. In addition, the neuroendocrine system played vital roles in the neuroendocrine-biomineralization crosstalk in metamorphosis. The regulation of biomineralization and settlement and morphology could be coupled with the same neuroendocrine signals.

## Additional files

Additional file 1: Kegg enrichment of differentially expressed genes in *P. f. martensii (XLSX 19 kb)*

Additional file 2: Gene Ontology enrichment of differentially expressed genes in *P. f. martensii (XLSX 22 kb)*

Additional file 3: The expression pattern of shell protein coding genes at different development stage in *C. gigas*. The y-axis is the normalized RPKM value. E, egg; TC, two cells; FC, four cells; EM, early morula; M, morula; B, blastula; RM, rotary movement; FS, free swimming;EG, early gastrula stage; G, gastrula; T1, trochophore 1; T2, trochophore 2; T3,trochophore 3; T4, trochophore 4; T5, trochophore 5; ED1, early D-larva 1; ED2, early D-larva 2; D1, D-larva 1; D2, D-larva 2; D3, D-larva 3; D4, D-larva 4; D5, D-larva 5; D6, D-larva 6; D7, D-larva 7; EU1, early umbo larva 1; EU2, early umbo larva 2; U1, umbo larva 1; U2, umbo larva 2; U3, umbo larva 3; U4, umbo larva 4; U5, umbo larva 5; U6, umbo larva 6; LU1, later umbo larva 1; LU2, later umbo larva 2; P1, pediveliger 1; P2, pediveliger 2; S, spat; and J, juvenile. (PDF 155 kb)

Additional file 4: Mantle tissue specific genes of *P. f. martensii (DOCX 15 kb)*

Additional file 5: Differentially expressed genes between early trochophore and trochophore stage involved in osteoclast differentiation in *P. f. martensii*. The red color represented the upregulation at trochophore stage, the green color represented the downregulation at trochophore stage. The yellow color represented the absence genes in *P. f. martensii*. (PDF 135 kb)

Additional file 6: Expression pattern of ECMs in shell matrix at different development stages in *C. gigas.* The y-axis is the normalized RPKM value. E, egg; TC, two cells; FC, four cells; EM, early morula; M, morula; B, blastula; RM, rotary movement; FS, free swimming;EG, early gastrula stage; G, gastrula; T1, trochophore 1; T2, trochophore 2; T3,trochophore 3; T4, trochophore 4; T5, trochophore 5; ED1, early D-larva 1; ED2, early D-larva 2; D1, D-larva 1; D2, D-larva 2; D3, D-larva 3; D4, D-larva 4; D5, D-larva 5; D6, D-larva 6; D7, D-larva 7; EU1, early umbo larva 1; EU2, early umbo larva 2; U1, umbo larva 1; U2, umbo larva 2; U3, umbo larva 3; U4, umbo larva 4; U5, umbo larva 5; U6, umbo larva 6; LU1, later umbo larva 1; LU2, later umbo larva 2; P1, pediveliger 1; P2, pediveliger 2; S, spat; and J, juvenile. (PDF 243 kb)

Additional file 7: The ecdysone receptors and the Nvds in bivalves. a. The ecdysone receptors in mollusk species. b, the expression pattern of Ecr at different development stages in *C. gigas*. E, egg; TC, two cells; FC, four cells; EM, early morula; M, morula; B, blastula; RM, rotary movement; FS, free swimming;EG, early gastrula stage; G, gastrula; T1, trochophore 1; T2, trochophore 2; T3,trochophore 3; T4, trochophore 4; T5, trochophore 5; ED1, early D-larva 1; ED2, early D-larva 2; D1, D-larva 1; D2, D-larva 2; D3, D-larva 3; D4, D-larva 4; D5, D-larva 5; D6, D-larva 6; D7, D-larva 7; EU1, early umbo larva 1; EU2, early umbo larva 2; U1, umbo larva 1; U2, umbo larva 2; U3, umbo larva 3; U4, umbo larva 4; U5, umbo larva 5; U6, umbo larva 6; LU1, later umbo larva 1; LU2, later umbo larva 2; P1, pediveliger 1; P2, pediveliger 2; S, spat; and J, juvenile. c. the expression pattern of Nvd at different development stages in *P. f. martensii* and *C. gigas*. Egg, egg; Fe, fertilized egg; B, blastula; G, gastrula; ET, early trochophore; T, trochophore; D, D-stage larvae; DF, D-stage larvae before feeding; EU, early umbo larvae; EL, eyed larvae; S, spat; J, juveniles. (PDF 772 kb)

Additional file 8: Expression pattern of acetylcholine receptors at different development stage in *P. f. martensii*. The y-axis is the normalized RPKM value. Egg, egg; Fe, fertilized egg; B, blastula; G, gastrula; ET, early trochophore; T, trochophore; D, D-stage larvae; DF, D-stage larvae before feeding; EU, early umbo larvae; EL, eyed larvae; S, spat; J, juveniles. (PDF 509 kb)

## Abbreviations

5-HT: 5-hydroxytryptamin
BMPs: Bone morphogenetic protein
CTR: Calcitonin receptor
CYP: Cytochrome P450
D: D-stage larvae
DA: Dopamine
ECMs: Extracellular matrixes
Ecr: Ecdysone receptor
EL: Eyed larvae
EPI: Epinephrine
ER: Estrogen receptor
ERK: Mitogen-activated protein kinase
ET: Early trochophore
EU: Early umbo larvae
NE: Norepinephrine
Nvd: Cholesterol 7-dehydrogenase
OPG: Tumor necrosis factor receptor superfamily member 11B
RANK: Tumor necrosis factor receptor superfamily member 11A
RUNKL: Tumor necrosis factor ligand superfamily member 11
S: Spat
smad: Mothers against decapentaplegic homolog
SMP: Shell matrix protein
SV2: Synaptic vesicle glycoprotein 2
T: Trochophore
TGF-beta: Transforming growth factor beta
ThR: Thyroid hormone receptor
TRAP: Tartrate-resistant acid phosphatase
VDR: Vitamin D3 receptor
VEGF: Vascular endothelial growth factor A
VWA: Von Willebrand factor A
