# Supplementary figures and images for: Developmental characteristics of pearl oyster Pinctada fucata martensii: insight into key molecular events related to shell formation, settlement and metamorphosis
Source: BMC Genomics. 2019 Feb 8;20:122. doi: 10.1186/s12864-019-5505-8 (PMC6368781; doi:10.1186/s12864-019-5505-8)

expression value

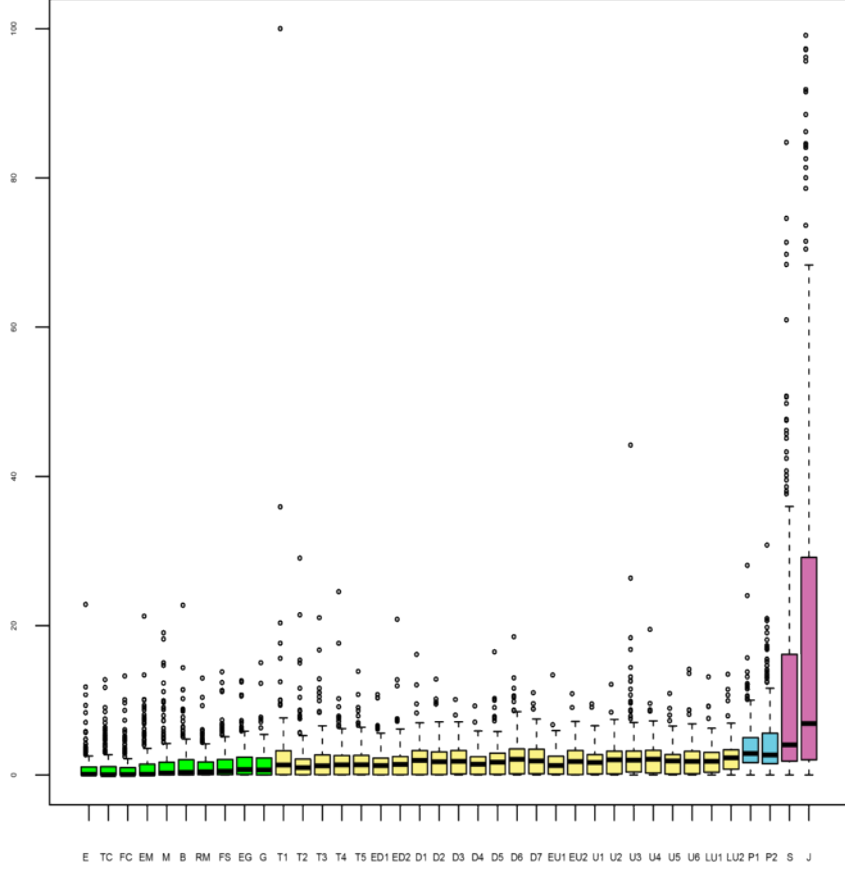

Supplement: Supplementary file 3 — The expression pattern of shell protein coding genes at different development stage in C. gigas. The y-axis is the normalized RPKM value. E, egg; TC, two cells; FC, four cells; EM, early morula; M, morula; B, blastula; RM, rotary movement; FS, free swimming;EG, early gastrula stage; G, gastrula; T1, trochophore 1; T2, trochophore 2; T3,trochophore 3; T4, trochophore 4; T5, trochophore 5; ED1, early D-larva 1; ED2, early D-larva 2; D1, D-larva 1; D2, D-larva 2; D3, D-larva 3; D4, D-larva 4; D5, D-larva 5; D6, D-larva 6; D7, D-larva 7; EU1, early umbo larva 1; EU2, early umbo larva 2; U1, umbo larva 1; U2, umbo larva 2; U3, umbo larva 3; U4, umbo larva 4; U5, umbo larva 5; U6, umbo larva 6; LU1, later umbo larva 1; LU2, later umbo larva 2; P1, pediveliger 1; P2, pediveliger 2; S, spat; and J, juvenile. (PDF 155 kb) [file 12864_2019_5505_MOESM3_ESM.pdf]

# OSTEOCLAST DIFFERENTIATION

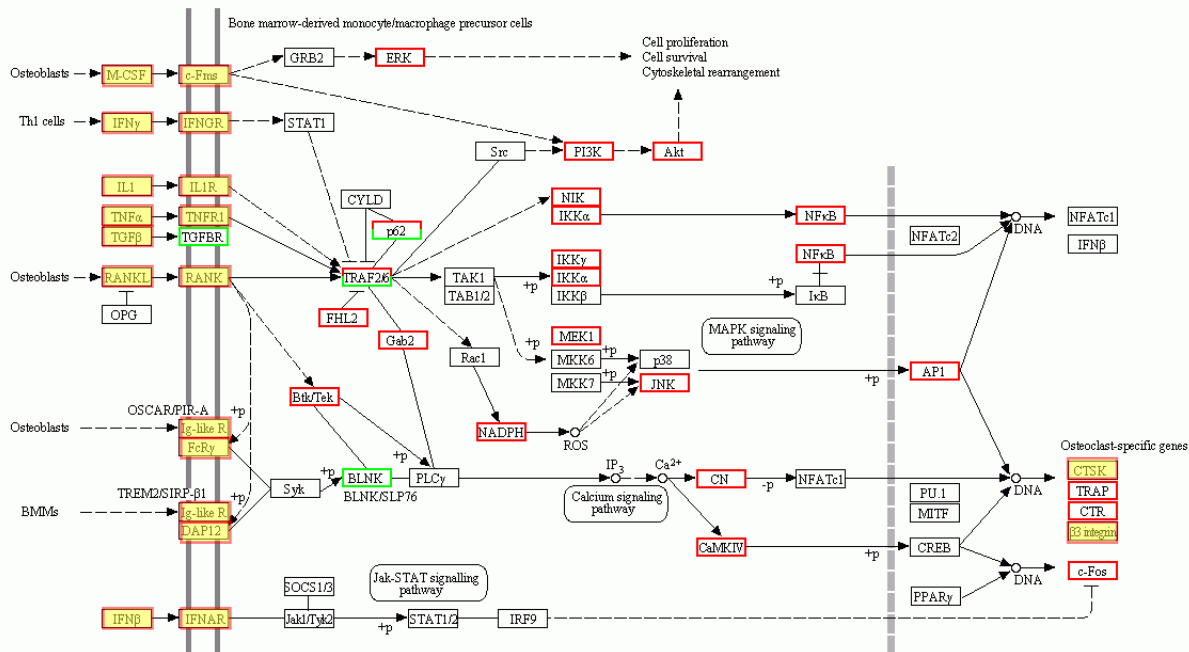

Supplement: Supplementary file 5 — Differentially expressed genes between early trochophore and trochophore stage involved in osteoclast differentiation in P. f. martensii. The red color represented the upregulation at trochophore stage, the green color represented the downregulation at trochophore stage. The yellow color represented the absence genes in P. f. martensii. (PDF 135 kb) [file 12864_2019_5505_MOESM5_ESM.pdf]

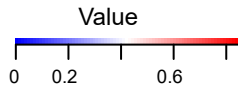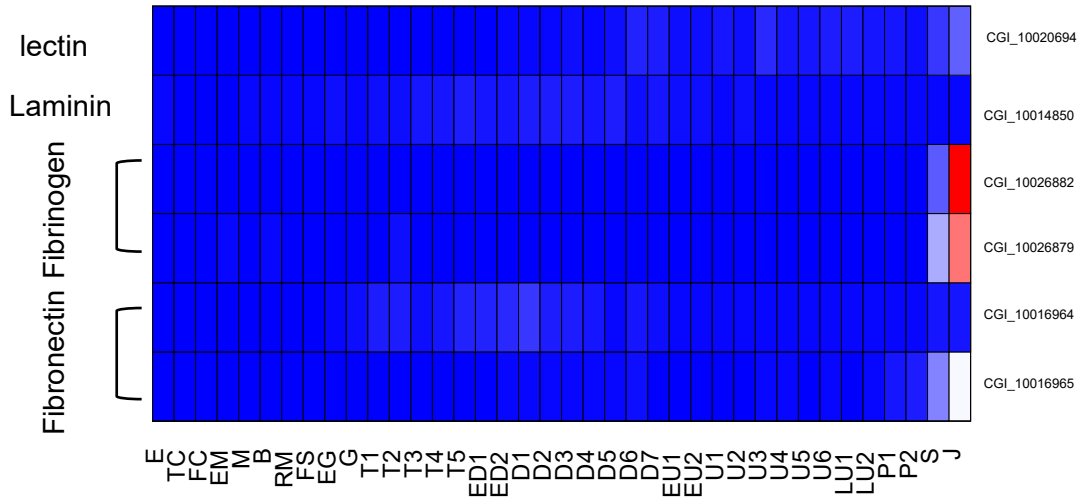

Supplement: Supplementary file 6 — Expression pattern of ECMs in shell matrix at different development stages in C. gigas. The y-axis is the normalized RPKM value. E, egg; TC, two cells; FC, four cells; EM, early morula; M, morula; B, blastula; RM, rotary movement; FS, free swimming;EG, early gastrula stage; G, gastrula; T1, trochophore 1; T2, trochophore 2; T3,trochophore 3; T4, trochophore 4; T5, trochophore 5; ED1, early D-larva 1; ED2, early D-larva 2; D1, D-larva 1; D2, D-larva 2; D3, D-larva 3; D4, D-larva 4; D5, D-larva 5; D6, D-larva 6; D7, D-larva 7; EU1, early umbo larva 1; EU2, early umbo larva 2; U1, umbo larva 1; U2, umbo larva 2; U3, umbo larva 3; U4, umbo larva 4; U5, umbo larva 5; U6, umbo larva 6; LU1, later umbo larva 1; LU2, later umbo larva 2; P1, pediveliger 1; P2, pediveliger 2; S, spat; and J, juvenile. (PDF 243 kb) [file 12864_2019_5505_MOESM6_ESM.pdf]

**a**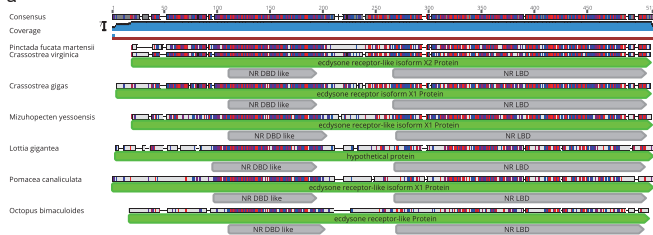**b**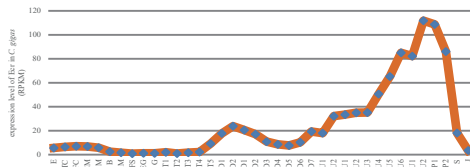**c**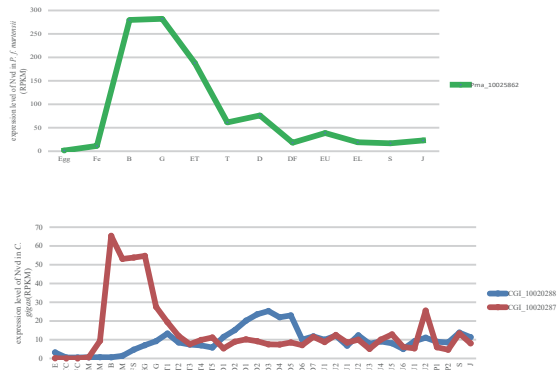

Supplement: Supplementary file 7 — The ecdysone receptors and the Nvds in bivalves. a. The ecdysone receptors in mollusk species. b, the expression pattern of Ecr at different development stages in C. gigas. E, egg; TC, two cells; FC, four cells; EM, early morula; M, morula; B, blastula; RM, rotary movement; FS, free swimming;EG, early gastrula stage; G, gastrula; T1, trochophore 1; T2, trochophore 2; T3,trochophore 3; T4, trochophore 4; T5, trochophore 5; ED1, early D-larva 1; ED2, early D-larva 2; D1, D-larva 1; D2, D-larva 2; D3, D-larva 3; D4, D-larva 4; D5, D-larva 5; D6, D-larva 6; D7, D-larva 7; EU1, early umbo larva 1; EU2, early umbo larva 2; U1, umbo larva 1; U2, umbo larva 2; U3, umbo larva 3; U4, umbo larva 4; U5, umbo larva 5; U6, umbo larva 6; LU1, later umbo larva 1; LU2, later umbo larva 2; P1, pediveliger 1; P2, pediveliger 2; S, spat; and J, juvenile. c. the expression pattern of Nvd at different development stages in P. f. martensii and C. gigas. Egg, egg; Fe, fertilized egg; B, blastula; G, gastrula; ET, early trochophore; T, trochophore; D, D-stage larvae; DF, D-stage larvae before feeding; EU, early umbo larvae; EL, eyed larvae; S, spat; J, juveniles. (PDF 772 kb) [file 12864_2019_5505_MOESM7_ESM.pdf]

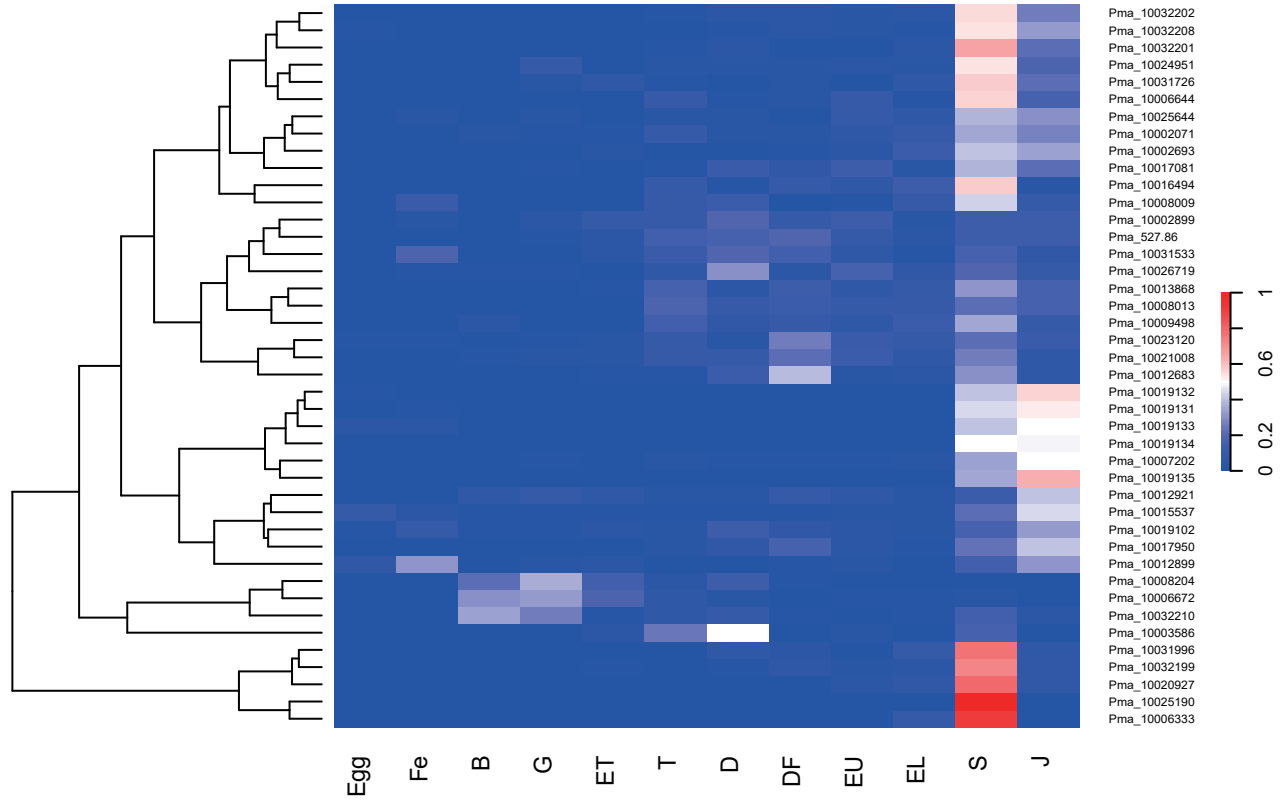

Supplement: Supplementary file 8 — Expression pattern of acetylcholine receptors at different development stage in P. f. martensii. The y-axis is the normalized RPKM value. Egg, egg; Fe, fertilized egg; B, blastula; G, gastrula; ET, early trochophore; T, trochophore; D, D-stage larvae; DF, D-stage larvae before feeding; EU, early umbo larvae; EL, eyed larvae; S, spat; J, juveniles. (PDF 509 kb) [file 12864_2019_5505_MOESM8_ESM.pdf]
