# Supplementary material for: Developmental characteristics of pearl oyster Pinctada fucata martensii: insight into key molecular events related to shell formation, settlement and metamorphosis
Source: BMC Genomics. 2019 Feb 8;20:122. doi: 10.1186/s12864-019-5505-8 (PMC6368781; doi:10.1186/s12864-019-5505-8)
Supplement: Supplementary file 4 — Mantle tissue specific genes of P. f. martensii (DOCX 15 kb) [file 12864_2019_5505_MOESM4_ESM.docx]

**Additional file 4. Mantle tissue specific genes of *P. f. martensii***

| MP | ME |
| --- | --- |
| Pma_146.639 | Pma_197.476 |
| Pma_191.168 | Pma_258.403 |
| Pma_233.205 | Pma_368.108 |
| Pma_522.510 | Pma_551.448 |
| Pma_10001008 | Pma_10000852 |
| Pma_10001161 | Pma_10002645 |
| Pma_10001162 | Pma_10002951 |
| Pma_10002287 | Pma_10002962 |
| Pma_10002519 | Pma_10004348 |
| Pma_10002747 | Pma_10006975 |
| Pma_10004648 | Pma_10009322 |
| Pma_10005159 | Pma_10010952 |
| Pma_10005178 | Pma_10011380 |
| Pma_10005329 | Pma_10012766 |
| Pma_10006224 | Pma_10013456 |
| Pma_10006779 | Pma_10013743 |
| Pma_10007160 | Pma_10014403 |
| Pma_10007421 | Pma_10015474 |
| Pma_10007422 | Pma_10015908 |
| Pma_10007794 | Pma_10016367 |
| Pma_10007841 | Pma_10016525 |
| Pma_10008043 | Pma_10017931 |
| Pma_10008044 | Pma_10018440 |
| Pma_10008261 | Pma_10018611 |
| Pma_10008262 | Pma_10018655 |
| Pma_10008951 | Pma_10018671 |
| Pma_10011438 | Pma_10019826 |
| Pma_10011661 | Pma_10021628 |
| Pma_10012960 | Pma_10021967 |
| Pma_10013219 | Pma_10023078 |
| Pma_10013921 | Pma_10024252 |
| Pma_10014348 | Pma_10026528 |
| Pma_10014474 | Pma_10026774 |
| Pma_10014699 | Pma_10026883 |
| Pma_10015641 | Pma_10027234 |
| Pma_10015642 | Pma_10028307 |
| Pma_10015664 | Pma_10028332 |
| Pma_10015665 | Pma_10029250 |
| Pma_10016377 | Pma_10029747 |
| Pma_10017299 | Pma_10030155 |
| Pma_10019560 | Pma_10030464 |
| Pma_10021718 | Pma_10030668 |
| Pma_10022629 | Pma_10030750 |
| Pma_10022630 | Pma_10032413 |
| Pma_10023307 | Pma_10032674 |
| Pma_10024220 | |
| Pma_10024855 | |
| Pma_10024933 | |
| Pma_10025727 | |
| Pma_10025915 | |
| Pma_10026127 | |
| Pma_10026227 | |
| Pma_10026678 | |
| Pma_10027763 | |
| Pma_10028422 | |
| Pma_10028517 | |
| Pma_10028755 | |
| Pma_10028756 | |
| Pma_10028757 | |
| Pma_10028879 | |
| Pma_10028881 | |
| Pma_10028883 | |
| Pma_10029492 | |
| Pma_10029532 | |
| Pma_10029740 | |
| Pma_10031979 | |
| Pma_10032249 | |
| Pma_10032252 | |
| Note: mantle pallium(MP), mantle edge (ME) | |
